# Anterior prominence of the femoral condyle varies among prosthesis designs and surgical techniques in total knee arthroplasty

**DOI:** 10.1186/s12891-021-04670-2

**Published:** 2021-09-12

**Authors:** Junya Itou, Umito Kuwashima, Masafumi Itoh, Ken Okazaki

**Affiliations:** Department of Orthopaedic Surgery, Tokyo Women’s Medical University, 8-1 Kawada-cho, Shinjuku-ku, 162-8666 Tokyo, Japan

**Keywords:** Total knee arthroplasty, Computer simulation, Patellofemoral overstuffing, Anterior reference, Posterior reference

## Abstract

**Background:**

Patellofemoral overstuffing after total knee arthroplasty (TKA) can cause limited range of motion and anterior knee pain. This study compared anterior prominence of femoral components among different prothesis designs in surgical simulation models utilizing the anterior reference (AR) and posterior reference (PR) techniques.

**Methods:**

Surgical simulations were performed using on a three-dimensional planning system preoperative computed tomography data of consecutive 30 patients with knee osteoarthritis scheduled to undergo TKA. Four implant models were used: Attune, Persona, Journey II, and Legion. Rotational alignment was set parallel to the transepicondylar axis and size was selected based on the absence of notch formation in the femoral anterior cortex and the best fit with the shape of the medial posterior femoral condyle. For each combination of surgical technique (AR or PR method) and implant model, measurements were taken of the maximum medial, central, and lateral prominence of the implant from the anterior femoral cortex.

**Results:**

Using either the AR or PR method, the medial and central prominences were significantly lower with Journey II than with the other models. The lateral prominence was the lowest with Attune in the AR method. The AR method was associated with significantly less prominence compared with the PR method, regardless of implant model.

**Conclusions:**

The degree of anterior prominence of the femoral implant is affected by the implant design when the AR method is used. The PR method is associated with greater anterior prominence compared with the AR method, and the pitch size is an additional factor in the PR method. Surgeons should be familiar with implant designs, including the thickness of the anterior flange and the available size selections.

## Introduction

Total knee arthroplasty (TKA) is widely known to provide good postoperative outcomes, with well-documented effectiveness in relieving pain and achieving good range of motion (ROM) [1–3]. Postoperative ROM is one of the factors associated with postoperative physical function [4] and patient satisfaction [5]. Various factors have been reported to influence postoperative ROM, including ligament balance, preoperative ROM, posterior condylar offset, and low anterior femoral condylar height [6, 7]. Recently, it has been reported that reducing the height of the anterior condylar after TKA can improve flexion [8]. In addition, anterior prominence of the femoral component has been suggested to influence postoperative ROM because it can cause overstuffing of the patellofemoral (PF) joint. However, this overstuffing is known to impair postoperative ROM [9] and can cause anterior knee pain [10].

Various factors have been associated with PF overstuffing, such as surgical technique and component design and size. Component upsizing has been associated with increased PF pressure during knee flexion in the swing phase of gait [11]. Differential model designs have been associated with differential PF pressure and patellar kinematics [12]. Even when adopting the same surgical technique, implant selection can result in different degrees of anterior femoral prominence because of both the design and pitch size of implants. However, this issue has not been focused on, and no information is available about the difference in anterior prominence of the femoral component among implant designs.

The objective of this study was to investigate differences in anterior prominence of the femoral components among different models when the prosthesis was implanted in the same patients with the same surgical techniques. To achieve this, we adopted a three-dimensional (3D) surgical simulation model to measure the anterior prominence of femoral condyle of several widely used prostheses placed with the anterior reference or posterior reference method.

We hypothesized that anterior prominence is minimized with implant models that have a thin anterior flange when using the anterior reference method or models that have a generous pitch size when using the posterior reference method.

The clinical relevance of this study is that the findings obtained would remind surgeons of the need to pay more attention to pitch size and design of the anterior flange in choosing or developing a new implant.

## Materials and methods

**Fig. 1:**
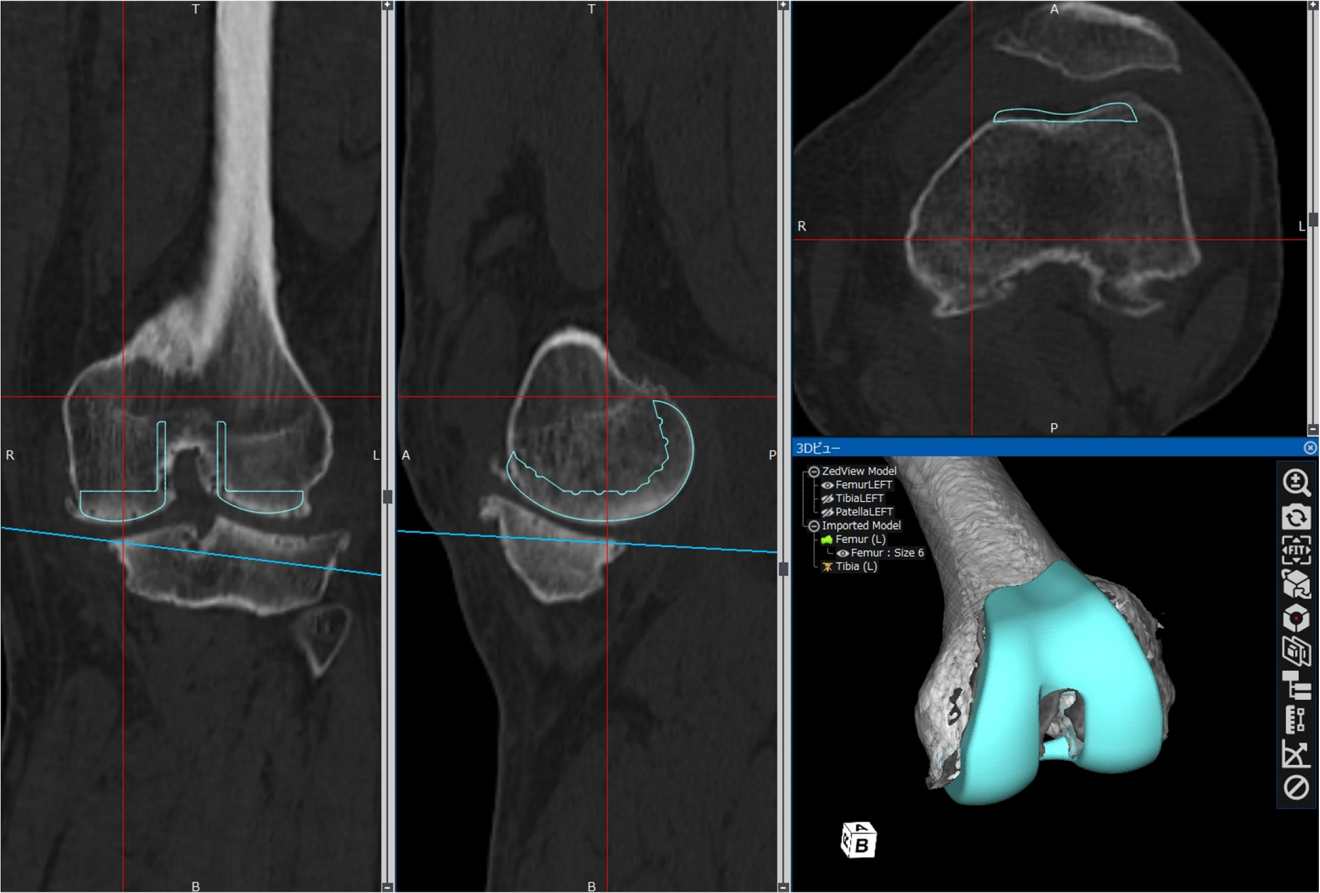
Representative implant placement simulation (Journey II, left knee osteoarthritis). Femoral implant is placed perpendicular to the three-dimensional functional axis for varus/valgus deformity and extension/flexion. Rotational alignment is set parallel to the transepicondylar axis (surgical epicondylar axis). Using the anterior reference method, the implant is placed so that the posterior of the anterior flange is in contact with the anterior cortex, and size selection is based on the best fit of the medial posterior condyle of the implant. With the posterior reference method, the implant is placed in contact with the posterior border of the medial posterior condyle, and the smallest size that did not form a notch in the anterior cortex is selected

**Fig. 2:**
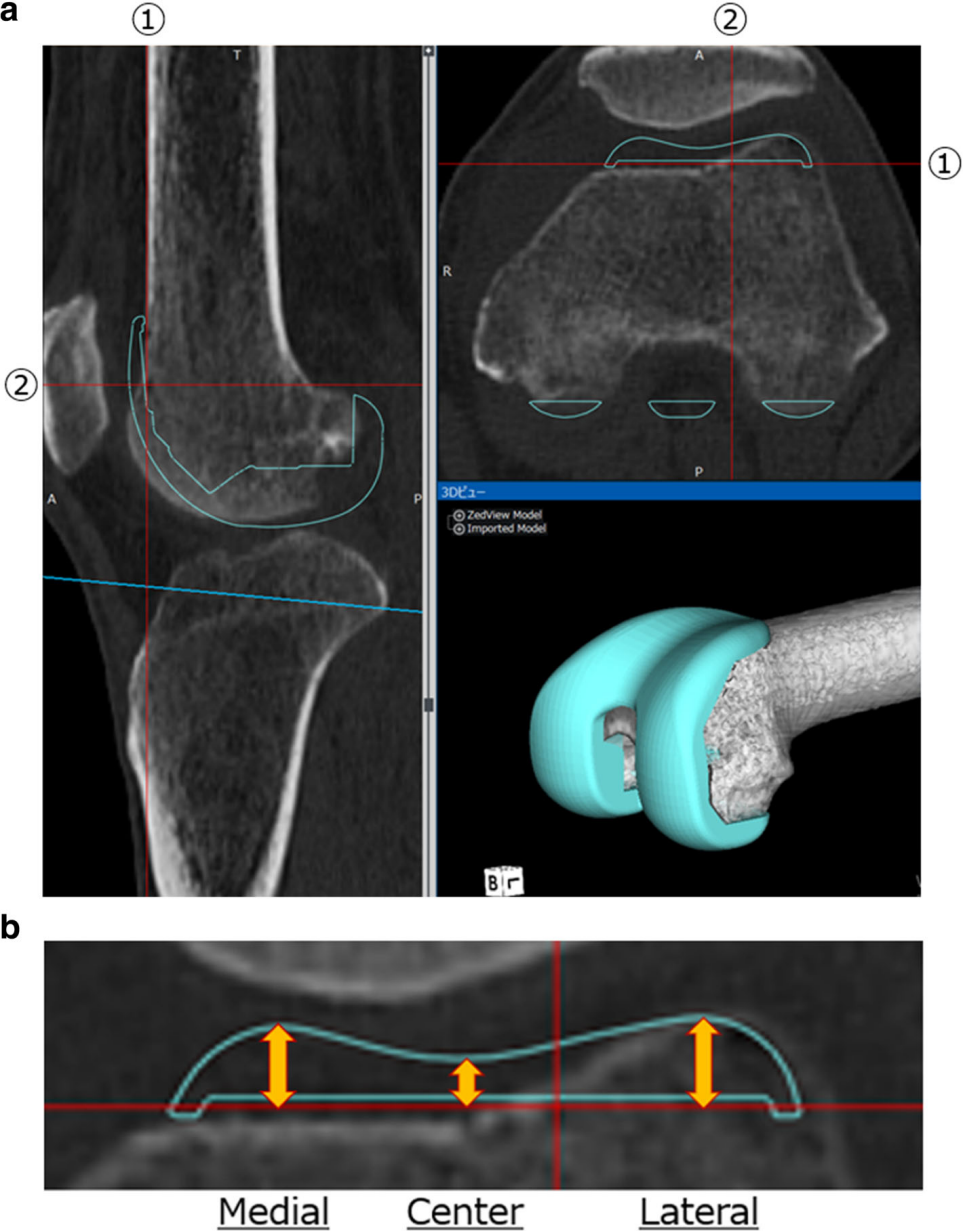
Details of measuring the anterior prominence. **a** The plane containing the anterior cortex and parallel to the surgical epicondyle axis was defined as line 1. **b** For each combination of surgical technique and implant model, measurements of the maximum medial, central, and lateral prominences of the implant from the line 1 are shown (double arrows)

Participants were 30 consecutive patients (12 men, 18 women; mean age 72.7 years, age range 53–85 years) with knee osteoarthritis (OA) scheduled to undergo TKA. In 11 patients, knees were classified as Kellgren-Lawrence Grade 3 and in 19 patients as Grade 4. Twenty-six patients had varus OA with a mean hip-knee-ankle angle of 170.9 ± 5.9° (156.0-179.0°), and 4 had valgus OA with a mean hip-knee-ankle angle of 185.2 ± 1.2° (184.2-186.6°) (Table 1). Surgical simulation was performed using preoperative computed tomography (CT) data on a 3D planning system (Zed Knee; LEXI, Tokyo, Japan). A femoral implant was placed perpendicular to the 3D mechanical axis for coronal and sagittal alignment. Rotational alignment was set parallel to the surgical transepicondylar axis (SEA). The distal anatomical axis of the femur was defined as the distal femoral intramedullary axis, which was marked between the centre of the knee and the centre of the intramedullary canal. Using the anterior reference method, the implant was placed so that the posterior of the anterior flange was in contact with the anterior cortex, and size was determined based on the best fit to the medial posterior condyle. The optimal size was selected, but a situation in which the implant was not in contact with the posterior border of the condyle was allowed in order to avoid notch formation. Using the posterior reference method, the implant was placed so that it was in contact with the posterior border of the medial posterior condyle, and the smallest size that did not form a notch in the anterior cortex was selected (Fig. 1). Four implant models were used: Attune PS (DePuy, Warsaw, IN; “Attune”); Persona PS (Zimmer-Biomet, Warsaw, IN; “Persona”); Journey II BCS (Smith & Nephew, Memphis, TN; “Journey II”); and Legion PS (Smith & Nephew; “Legion”). The plane containing the anterior cortex and parallel to the surgical epicondyle axis was defined as line 1 (Fig. 2a). For each combination of surgical technique and implant model, the maximum medial, central, and lateral prominence of the implant from line 1 (mm; double arrows) and the component size were measured (Fig. 2b).

**Table 1:** Demographic data of 30 patients with knee osteoarthritis scheduled to undergo total knee arthroplasty

Age (years)	72.7 ± 6.9 (53–85)
Sex (male / female)	12 / 18
BMI (kg/m^2)^	26.6 ± 4.6 (18.0 to 36.5)
Affected side (right / left)	15 / 15

### Statistical analysis

The Steel–Dwass test was used for multiple comparisons between the four implant groups; Wilcoxon’s signed-rank test was used for paired comparison of the anterior reference versus posterior reference method with the same implants. Statistical significance was based on p-values of less than 5 %. Statistical analysis was performed using JMP software (SAS, Cary, NC).

For the measurement of intra-observer reliability, the maximum medial, central, and lateral prominences were measured twice at an interval of ≥ 14 days for each implant model in 4 randomly selected patients. The resulting intraclass correlation coefficient (ICC 1,1) was 0.94. In a statistical power analysis, a power of 0.8, alpha of 0.05, and standard deviation of 0.5 were assumed. Based on the results of a pilot study, the effect size was set to detect differences in prominence between models of 0.5 mm, 0.5 mm, and 1 mm. Finally, the sample size was calculated as 27.

### Ethical approval and consent to participate

All procedures involving human participants were in accordance with the ethical standards of the 1964 Helsinki Declaration and its later amendments. This study was conducted with approval from the ethics committees of Tokyo Women’s Medical University (approval no. 4578, December 12, 2017). Informed consent was obtained via an opt-out procedure.

## Results

The anatomical parameters used for implant placement in the surgical simulation for 30 patients are listed in Table 2. Mean valgus angle against the distal anatomical axis of the femur was 6.3 ± 1.9 and mean external rotation angle from the posterior condylar axis was 3.8 ± 1.6°. The mean sagittal plane angle against the distal bone axis was 1.3 ± 1.9° extension.

**Table 2:** Anatomical parameters

Valgus (°)	6.3 ± 1.9 (2.6 to 10.5)
External rotation^a^(°)	3.8 ± 1.6 (0.5 to 7.5)
Sagittal alignment^b^(°)	−1.3 ± 1.9 (− 5.5 to 2.2)

^a^Expressed as an external rotation angle from the posterior condylar angle

^b^Negative values indicate extension and positive values indicate flexion against the distal bone axis

**Fig. 3:**
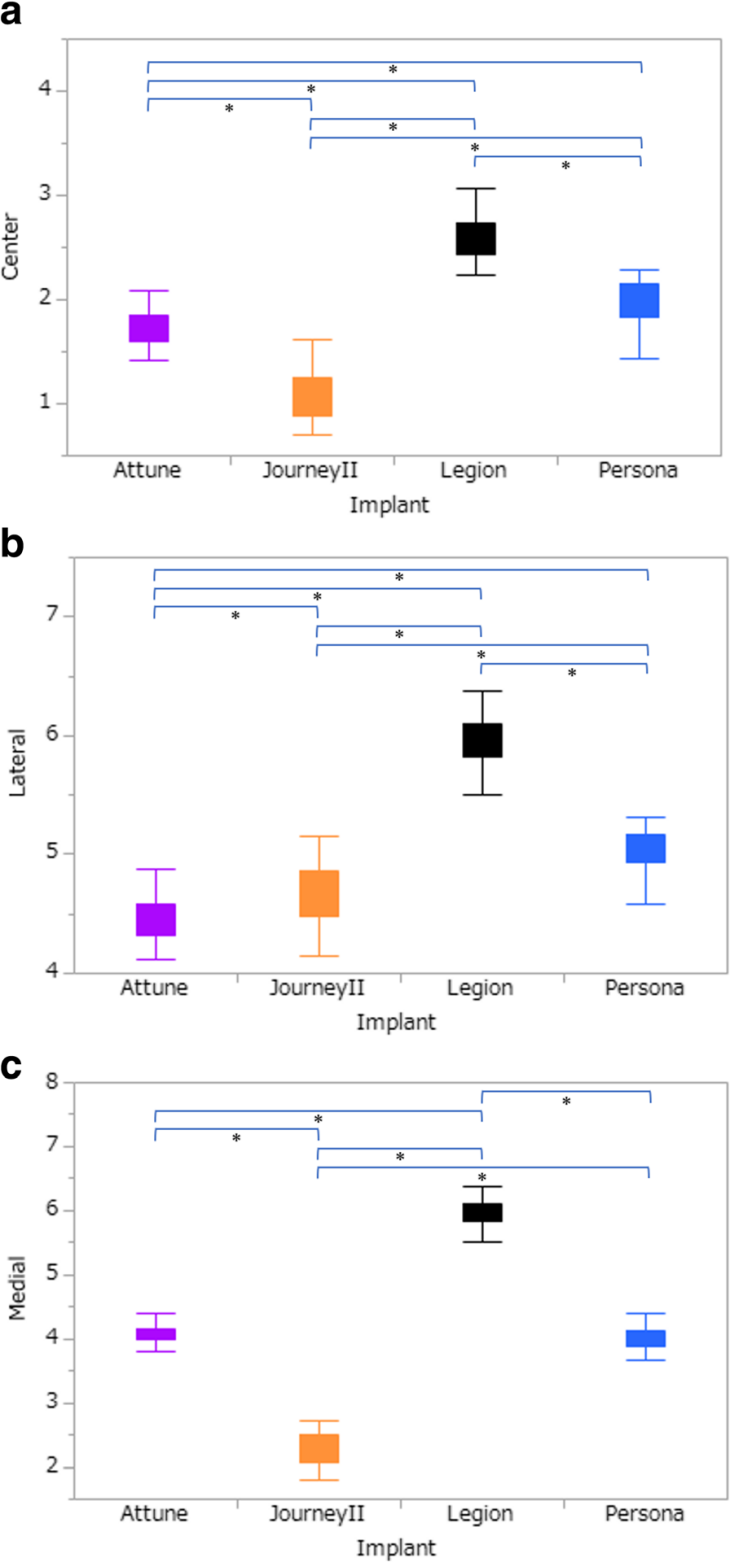
Comparison of medial, central, and lateral prominences (mm) using the anterior reference method with different implants. *Steel–Dwass test. The medial and central prominences were significantly lower with Journey II than with the other models (*p* < 0.001). The medial prominences were significantly lower with Attune and Persona than with Legion, but there was no significant difference between Attune and Persona. The central prominences were significantly lower in the order of Journey II, Attune, Persona, and Legion. The lateral prominence was significantly lower with Attune than with the other models (*p* < 0.04). The lateral prominences were significantly lower in the order of Attune, Journey II, Persona, and Legion

With the anterior reference method, the medial and central prominences were significantly lower with Journey II than with the other models (*p* < 0.001, Fig. 3). In addition, the medial prominences were significantly lower with Attune and Persona than with Legion, but there was no significant difference between Attune and Persona. Furthermore, the central prominences were significantly lower in the order of Journey II, Attune, Persona, and Legion. The lateral prominence was significantly lower with Attune than with the other models (*p* < 0.04, Fig. 3). In addition, the lateral prominences were significantly lower in the order of Attune, Journey II, Persona, and Legion.

**Fig. 4:**
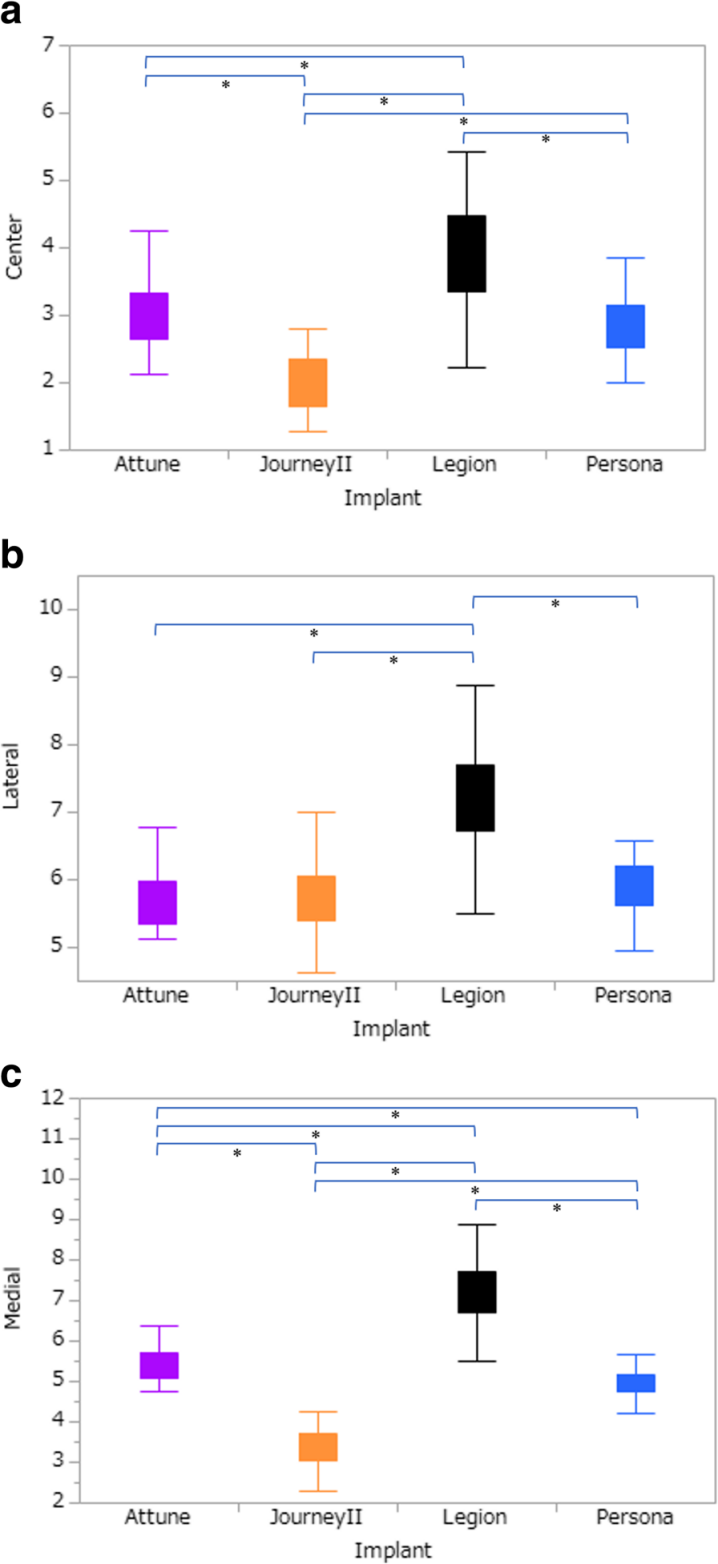
Comparison of medial, central, and lateral prominences (mm) using the posterior reference method with different implants. *Steel–Dwass test. The medial and central prominences were significantly lower with Journey II than with the other models (*p* < 0.0001). The medial prominences were significantly lower in the order of Journey II, Persona, Attune, and Legion. Furthermore, the central prominences were significantly lower with Attune and Persona than with Legion, but there was no significant difference between Attune and Persona. Lateral prominence was significantly lower with Attune, Journey II, and Persona than with Legion (*p* < 0.0001), with no significant differences among the Attune, Journey II, and Persona models (*p* > 0.21)

With the posterior reference method, the medial and central prominences were significantly lower with Journey II than with the other models (*p* < 0.0001, Fig. 4). In addition, the medial prominences were significantly lower in the order of Journey II, Persona, Attune, and Legion. Furthermore, the central prominences were significantly lower with Attune and Persona than with Legion, but there was no significant difference between Attune and Persona. Lateral prominence was significantly lower with Attune, Journey II, and Persona than with Legion (*p* < 0.0001, Fig. 4), with no significant differences among the Attune, Journey II, and Persona models (*p* > 0.21, Fig. 4).

With the posterior reference method, component upsizing was needed to avoid anterior notch formation in 4, 3, 5, and 8 cases placed with Attune, Persona, Journey II, and Legion, respectively.

**Fig. 5:**
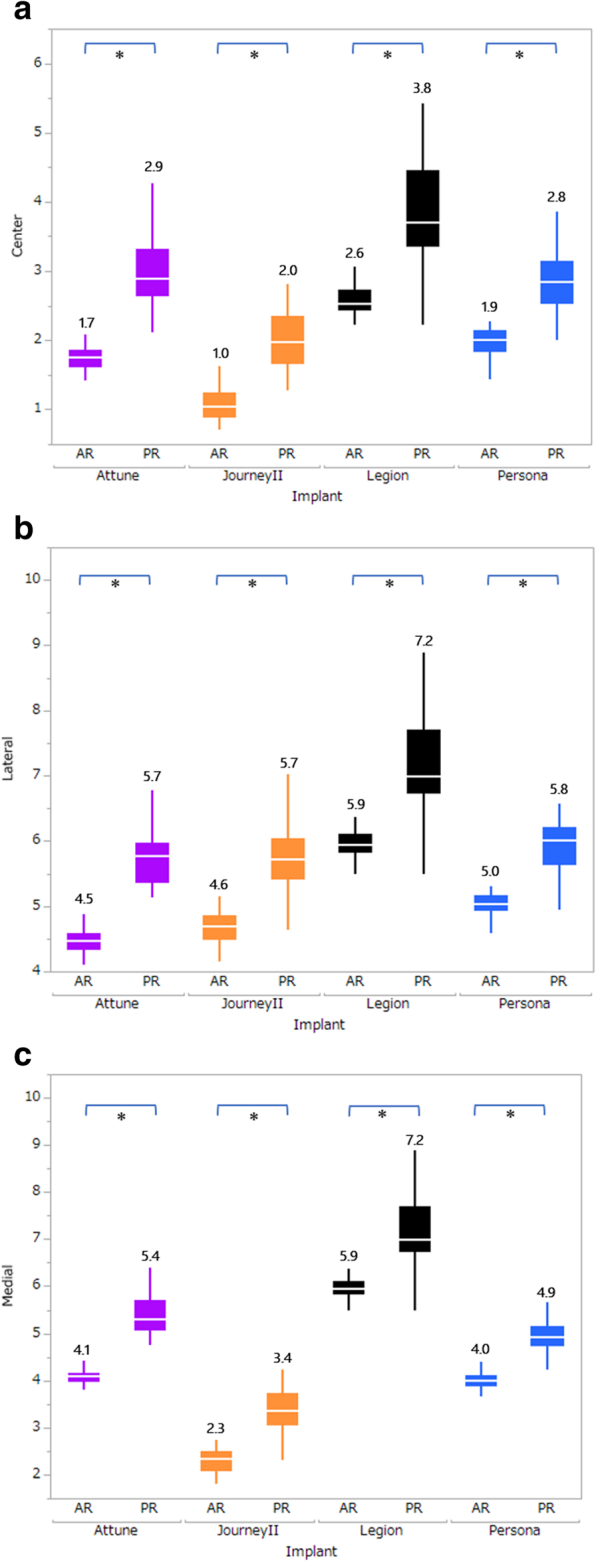
Comparison of anterior and posterior reference methods. *Wilcoxon’s signed-rank test. For all implants, the anterior reference method was associated with significantly lower anterior prominence (*p* < 0.05)

For each implant model, the medial, central, and lateral prominences were significantly lower using the anterior reference method than the posterior reference method (*p* < 0.05, Fig. 5).

## Discussion

The most important finding of this study was that the anterior prominence of the femoral condyle varied significantly depending on the implant design in either the anterior reference or posterior reference method. Journey II provided the least medial and central prominence among the 4 designs investigated. The anterior flange of Journey II is thinner than that of other models, suggesting the design influences the results significantly. In the posterior reference method, Attune and Persona were also associated with lower anterior prominence, probably because the generous pitch sizes of these models allowed for the selection of the size most closely attached to the anterior cortex.

Anterior prominence of the femoral component can lead to PF overstuffing and can affect ROM [9]. Nishitani et al. [8] have reported that a decrease in the height of the anterior condyle from the original level is associated with increased postoperative flexion. Relatively good clinical outcomes in terms of postoperative ROM have been reported with Journey II. Brilhault et al. reported a mean ROM of 124 ± 9.8° [1]. Vascellari et al. described improved mean ROM from 99.3° preoperatively to 119° postoperatively [13], and Taniguchi et al. reported a corresponding improvement from 117.7° (SD15.3°) to 129.0° (SD 9.6°) [14]. Although these reports did not compare Journey II with other models under the same conditions, the relatively good ROM results reported with Journey II may be partially attributable to lower anterior femoral prominence than with other models. Selecting an implant with a thin flange may be a good strategy for gaining better postoperative ROM. In the anterior reference method, the degree of anterior prominence of the implant is likely affected by the implant design.

In contrast, using the posterior reference method, other factors are suspected to be involved. When an implant has been selected based on the best fit to the shape of the medial condyle, upsizing would be needed if an anterior notch is formed. In such cases, the use of models with generous pitch sizes is likely to result in a smaller mean anterior prominence. In fact, relatively small numbers of patients treated with Persona or Attune required implant upsizing, likely because of the generous pitch size and design of these models. Persona has a pitch size of 2 mm and Journey II and Attune have a pitch size of 3 mm. In contrast, Legion was associated with higher anterior prominence, likely due to its design and pitch size. More specifically, its pitch size of 4 mm and greater flange thickness compared with the other models appeared greatly impact on the outcome.

The decision to select the anterior reference or posterior reference method in an actual operation depends on the mechanical property of the implant and the surgical technique to be used. It is generally understood that when using the anterior reference method, notch formation can be avoided, but it is associated with difficulty in adjusting the posterior condylar offset and flexion gap. In contrast, the posterior reference method is associated with easier adjustment of the posterior condylar offset and flexion gap, but with higher risks of notch formation and PF overstuffing [15]. The present study did not take into account compatibility to the size of tibial implant component or the flexion gap. Therefore, in actual surgery, surgeons should be cautious in selecting surgical techniques and procedures. Nevertheless, the fact that Journey II provided less anterior prominence in either the anterior reference or posterior reference method suggests that the implant design significantly affects it whichever method is to be used.

In this study, we defined implant placement on the coronal and sagittal planes perpendicular to the 3D functional axis. The mean valgus angle was 6.3°, which is comparable to that reported previously in a study using 3D-CT (5.4 ± 0.7°) [16] and in another study (6.3° when limited to knees with severe genu varum) [17]. The transepicondylar axis (TEA) can be either the clinical epicondylar axis or SEA. In this study, the SEA was used based on previous CT-based studies [18, 19]. Meric et al. [20] defined the SEA as externally rotated by a mean of 3.3 ± 1.5° from the posterior condylar axis in a CT analysis of 13,546 knees. Victor [21] has concluded that the SEA is externally rotated by a mean of 3° from the posterior condylar axis based on a review of rotation data. These values are consistent with corresponding values obtained from the patients analyzed in this study. Mean implant angle on the sagittal plane against the distal femoral axis was 1.3 ± 1.9° extension, which was considered to be prone to notch formation. However, this was unlikely to affect the results of the present study because differences between implants were investigated using surgical simulation under the same condition of placing the implant perpendicular to the 3D functional axis.

There are several limitations to this study. First, we evaluated only 4 implant models. In this study, we sought to learn how implant design and sizing affect the anterior prominence rather than to search for the best implant available, and we selected models that are representative of the most commonly used models from each manufacturer in the registry. It is recommended that surgeons pay greater attention to the design of anterior flange of the implant they use. Second, the femoral external rotation angle was only set against the SEA. We also evaluated all cases by setting the external rotation angle as being externally rotated by 3° from the posterior condylar line and obtained similar results. Therefore, only the data aligned to the SEA was presented to avoid confusion. Third, mediolateral overhang of the femoral implant, was not taken into account, particularly in the posterior reference method. However, because the main purpose of this study was to evaluate the degree of anterior prominence of the femoral implant, further studies on the details of overhang are warranted.

## Conclusions

The degree of anterior prominence of the femoral implant is affected by implant design when the anterior reference method is used. The posterior reference method is associated with greater anterior prominence compared with the anterior reference method, with pitch size identified as an additional influencing factor. Journey II is associated with the least anterior prominence when using either method. The various implant designs were shown to be associated with different degrees of anterior prominence, so additional attention is needed to the effects of surgical techniques and pitch size. In terms of relevance to daily clinical practice, our findings highlight the need for surgeons to be familiar with implant designs, including the thickness of the anterior flange and available size selections, to ensure favorable postoperative outcomes.

## Data Availability

The datasets used and/or analysed during the current study are available from the corresponding author upon reasonable request.

## Abbreviations

CT: Computed tomography
TKA: Total knee arthroplasty
PF: Patellofemoral
ROM: Range of motion
